# Low Expression of Claudin-7 as Potential Predictor of Distant Metastases in High-Grade Serous Ovarian Carcinoma Patients

**DOI:** 10.3389/fonc.2020.01287

**Published:** 2020-08-04

**Authors:** Chiara Romani, Valentina Zizioli, Marco Silvestri, Laura Ardighieri, Mattia Bugatti, Michela Corsini, Paola Todeschini, Sergio Marchini, Maurizio D'Incalci, Laura Zanotti, Antonella Ravaggi, Fabio Facchetti, Angela Gambino, Franco Odicino, Enrico Sartori, Alessandro Davide Santin, Stefania Mitola, Eliana Bignotti, Stefano Calza

**Affiliations:** ^1^Department of Molecular and Translational Medicine, University of Brescia, Brescia, Italy; ^2^Division of Obstetrics and Gynecology, ‘Angelo Nocivelli’ Institute of Molecular Medicine, University of Brescia and ASST-Spedali Civili of Brescia, Brescia, Italy; ^3^Division of Obstetrics and Gynecology, ASST Spedali Civili di Brescia, Brescia, Italy; ^4^Biomarkers Unit, Department of Applied Research and Technological Development, Fondazione IRCCS Istituto Nazionale dei Tumori di Milano, Milan, Italy; ^5^Unit of Biostatistics and Bioinformatics, Department of Molecular and Translational Medicine, University of Brescia, Brescia, Italy; ^6^Department of Pathology, ASST Spedali Civili of Brescia, Brescia, Italy; ^7^Department of Oncology, IRCCS, “Mario Negri” Institute for Pharmacological Research, Milan, Italy; ^8^Division of Obstetrics and Gynecology, Department of Clinical and Experimental Sciences University of Brescia, Brescia, Italy; ^9^Department of Obstetrics, Gynecology and Reproductive Sciences, Yale University School of Medicine, New Haven, CT, United States; ^10^Big & Open Data Innovation Laboratory, University of Brescia, Brescia, Italy

**Keywords:** high grade serous ovarian carcinoma, fallopian tube epithelium, claudins, distant metastases, hematogenous spread

## Abstract

High-grade serous ovarian carcinoma (HGSOC) usually spreads directly into the peritoneal cavity following a transcoelomic dissemination route, although distant hematogenous metastasis exist and have been reported. However, no tumor markers can currently predict the risk of distant metastases in HGSOC. Claudins, belonging to tight-junction proteins, are dysregulated in HGSOC and functionally related to cancer progression. Here we analyzed claudin-3, -4, and -7 expression as potential markers of distant metastases. Using quantitative RT-PCR and immunohistochemistry we assessed the expression of claudins in primary HGSOC tissues, normal ovarian, and normal fallopian tube epithelia and correlated it with clinicopathological features, including the site of metastasis and the route of dissemination. Gene set enrichment analysis was performed on microarray-generated gene expression data to investigate key pathways in patients with distant metastases. We found the overall expression level of claudin-3, -4, and -7 mRNA decreased in HGSOC compared to normal tubal epithelium, currently considered the potential site of origin of many HGSOC. The reduced expression of claudin-7 is significantly associated with the development of distant metastases (*p* = 0.016), mainly by hematogenous route (*p* = 0.025). In patients with diminished expression of claudin-7, immunohistochemical staining revealed a heterogeneous pattern of membranous staining with discontinuous expression of claudin-7 along the cell border, indicative of a dischoesive architecture. The estimated reduction in the probability of distant disease is of 39% per unit increase in the level of claudin-7 (*p* = 0.03). Genes involved in epithelial to mesenchymal transition, hypoxia, and angiogenesis processes resulted strongly associated to hematogenous recurrence. Our data suggest a potential role of claudin-7 in discriminating distant metastatic events in HGSOC patients. The quantification of its expression levels could be a useful tool to identify patient deserving a personalized follow-up in terms of clinical and radiological assessment.

## Introduction

High-grade serous carcinoma (HGSOC) is the most frequent and aggressive ovarian cancer, commonly associated with a poor outcome. More than 70% of patients present with advanced disease at diagnosis, with widespread metastases within the peritoneal cavity, and the majority of them experience recurrence after primary treatment (1). Although the intra-abdominal dissemination is predominant, distant metastases occur in 8–10% of patients at the time of diagnosis and in 20% of patients at recurrence (2). Survival after distant metastases is usually very poor (3). Current clinicopathological prognostic factors are not adequate to predict distant spread of the disease and no molecular markers have been shown to be associated with distant metastatic behavior in HGSOC.

The lack of consensus on the ovarian cancer site of origin, which negatively impact on screening strategies, can partially explain HGSOC late diagnosis. Recent insights suggest that HGSOC might arise both from ovarian surface mesothelium and distal fallopian tube epithelium. In line with this new paradigm, a large percentage of HGSOC is thought to originate in the fallopian tubes and secondarily involving the ovary and eventually the peritoneal cavity (4).

Claudins are major components of the apical tight junction complex, which is responsible for adhesion and selective permeability of epithelial cells. The 27 known members of the claudin family are differently expressed in a tissue-specific manner and most cell types simultaneously express several different claudins (5). Beside their barrier function, mounting evidence suggests a direct role in regulating cell survival and proliferation, and in promoting cell migration and invasion possibly through epithelial-to-mesenchymal transformation (EMT) (6).

Claudin-3, -4, and -7 are the most frequently dysregulated members of this family in human epithelial cancers and their functional importance in cancer progression to metastatic disease is well-established (7). Loss of claudin-7 has been reported to correlate with venous invasion and liver metastases in colorectal cancer (8). Che et al. reported that a decreased expression of claudin-3 significantly correlate with lymph node metastases, disease recurrence, and shorter overall survival in squamous cell lung carcinoma (9), whereas downregulation of claudin-4 in gastric cancer correlates with tumor aggressiveness and poor survival (10).

In sharp contrast to the notion that the loss of cell-cell junctional adhesion support the metastatic abilities of cancer cells, overexpression of claudins has been also linked to tumor progression, as in prostate cancer, where claudin-3 and -4 upregulation was consistently identified in clinically advanced tumors and positively correlated with disease recurrence (11).

In ovarian cancer, elevated claudin-3, -4, and -7 expression has been shown compared to normal ovary in multiple independent studies, supporting the conclusion that claudins are upregulated during malignant transformation (12, 13).

In the present study using quantitative PCR (RT-qPCR) and immunohistochemistry we evaluated the expression of claudin-3, -4, and -7 in a large cohort of HGSOC tumors, using fallopian tube epithelium and ovarian surface mesothelium as positive and negative controls, respectively. Claudin mRNA and protein expression was correlated with HGSOC clinicopathological characteristics, focusing on site of metastases and pattern of dissemination. The predictive power of claudins in discriminating patients with an increased likelihood of developing distant disease was also investigated.

## Materials and Methods

### Patient Information

This study was performed on 105 HGSOC patients diagnosed and treated at the Division of Gynecologic Oncology of the University of Brescia (Italy) between 2002 and 2015. Normal luminal fallopian tube epithelium and normal ovarian surface mesothelium were obtained from patients undergoing surgery for benign pathologies at the same Institution. The study was performed following the Declaration of Helsinki set of principles and approved by the Research Review Board-the Ethic Committee- of the ASST Spedali Civili, Brescia, Italy (study reference number: NP1676). Written informed consent was obtained from all patients enrolled. Information regarding age, stage, macroscopic residual tumor after cytoreductive surgery (RT), location, and pattern of spread of recurrence were recorded.

According to the International Federation of Gynecologic Oncology (FIGO) staging system (14), patients were classified as: (i) stage III, with extra-pelvic peritoneal involvement, with or without positive retroperitoneal lymph nodes (ii) stage IV, including patients with distant metastases, with a distinction between IVA, with malignant pleural effusion, and IVB with parenchymal liver/splenic metastases and extra-abdominal metastases such as brain, bones, lung, breast, extra-abdominal lymph nodes, and skin.

Cancer recurrence or progression were defined according to the platinum-free interval (PFI), following RECIST 1.1 criteria (15). According to location, recurrences were classified as: (i) abdominal, based on lesions evaluable by imaging in the intra- and extrapelvic peritoneum, including recurrence in retroperitoneal lymph nodes, such as pelvic and para-aortic lymph nodes; (ii) distant, including recurrence in the liver parenchyma, lung, bone, and brain confirmed by CT or MRI scan.

According to the pattern of spread, recurrences were classified as: (i) transcoelomic, defined by the presence of metastases in the peritoneum of the pelvis and abdominal area; (ii) lymphatic, including recurrence to pelvic and para-aortic lymph nodes, as well as distant lymph nodes; (iii) hematogenous, defined as cases with metastatic lesions in bones, liver, lung, and brain parenchyma; (iv) mixed, if concomitant pattern of spread were observed.

### Tissue Collection and Processing

HGSOC tissues were obtained from chemotherapy-naïve patients at the time of primary surgery, snap-frozen in liquid nitrogen within 30 min and stored at −80°C until processing. Tumor content was assessed by H&E staining on frozen sections. Only samples containing at least 70% of tumor epithelial cells, as assessed by a staff pathologist, were used for further RNA extraction.

Twenty-six normal luminal fallopian tube and 14 normal ovarian surface mesothelium (Hose) were collected by scraping, as described (16). RNA was extracted using TRIZOL reagent (Life Technologies), purified using RNeasy MiniElute Cleanup kit (Qiagen) and further treated with TURBO DNase enzyme (Ambion, Applied Biosystems) to remove the contaminating DNA eventually present. RNA quantity was evaluated spectrophotometrically, and the quality was assessed with the Agilent 2100 bioanalyzer (Agilent technologies Inc.). Only samples with good RNA yield and no RNA degradation (28S:18S >1.8 and RNA integrity >8.5) were retained for further experiments (Supplementary Figure 1). For Hose, RNAs were pooled together to obtain a sufficient quantity to carry out the experiments. Finally, 9 samples of Hose were obtained from 14 patients.

### Quantification of Claudin-3, -4, and -7 mRNA by RT-qPCR

cDNA was generated from total RNA using the SuperScriptII reverse transcriptase (Invitrogen). Quantitative real-time PCR (RT-qPCR) for claudins and the reference genes HPRT1 and PPIA was established in a multiplex procedure for simultaneous amplification of each template, as described by our group (17). The 2^−ΔΔCt^ method was applied to calculate claudin relative expression. TaqMan Gene Expression Assay for claudin-3 (ID: Hs00265816_s1), -4 (ID: Hs00976831_s1), and -7 (ID: Hs00600772_m1) was obtained from Applied Biosystems as Assay-on-Demand products.

### Immunohistochemistry

Four-micron sections from formalin-fixed, paraffin-embedded (FFPE) tissues were stained for claudin-7 after antigen retrival by microwave treatment in EDTA buffer-pH 8.0, applying anti-Claudin-7 (mouse, clone 5D10F3, 1:80, Thermo Scientific) for 60 min. The reaction was revealed using Novolink Polymer (Leica Microsystem) or Envision System-HRP Labeled Polymer anti-Mouse (Dako) followed by DAB.

Immunoreactivity was evaluated by four independent observers (CR, LA, MB, EB); staining was graded for intensity (1–3+) and percentage of stained cells, and a single H score was obtained by multiplying the intensity and the percentage staining. Positivity was defined by at least 1+ intensity in 10% or more of the cells (H-score = 10).

### Gene Expression Profiling

Gene expression profile of HGSOC tissues was generated using G4851B Agilent SurePrint G3 Human gene expression 8 × 60K microarray (Agilent Technologies) according to the manufacturer's protocol. Briefly, 100 ng of total RNA was converted to cDNA, followed by *in vitro* transcription and Cy3-CTP labeling. After fragmentation, the labeled cRNA was hybridized to the microarrays and scanned, as described in the manufacturer's protocol. The intensity data were extracted using the Feature Extraction Software v11 (Agilent Technologies). In accordance to the MIAME guidelines, the array data files have been uploaded to the EMBL-EBI Arrayexpress repository (https://www.ebi.ac.uk/arrayexpress/experiments/E-MTAB-7083/ and https://www.ebi.ac.uk/arrayexpress/experiments/E-MTAB-7084/).

### Statistical Analysis

RT-qPCR values were modeled on log scale. Comparison of RT-qPCR expression between Hose, tube, and HGSOC tissues were performed using *t*-test for independent and reported as Fold Change (FC, the ratio between group medians) and corresponding confidence interval (CI 95%). The correlation between claudin-3, -4, and -7 expression was computed using Pearson correlation coefficient.

An overall score of claudin expression was calculated using principal component analysis (PCA), a well-known algorithm which projects data onto an orthogonal set of variables that subsequently capture the most variance in the data.

The relationship between claudin expression and clinicopathologic parameters was evaluated using univariate and multivariate linear models. To evaluate the potential predictive role of claudins we fitted logistic regression models for each claudin. Model performance was evaluated using bias adjusted C-index computed via bootstrap (B = 200), with its correspondent 95% confidence interval.

All analysis were performed using R (version 3.5.0) and assuming two sided significance level of 5%.

### Microarray Data Processing and Gene Set Enrichment Analysis

Raw expression data were pre-processed first applying a background correction (18), followed by a quantile normalization between arrays (19).

Gene Set Enrichment analysis was performed using GSEA (20) on the gene list ranked according to a moderated *t*-statistics (21), comparing patients with hematogenous and transcoelomic recurrence.

GSEA analysis is a statistical method for calculating the enrichment of a gene list in a specific pathway. Briefly, all the genes in a particular gene list are ranked based on a particular metrics (*t*-statistic) in order to derive an Enrichment Score (ES) that reflects the degree to which a pathway is overrepresented at the top or bottom of the ranked list of genes. Gene sets collection including canonical pathways and signatures from the literature (C2) and specific well-defined biological states or processes (HALLMARK) from the MSigDB database (http://www.broadinstitute.org/gsea/msigdb) were tested for enrichment. The gene sets with *P* < 0.01 were considered significantly enriched.

## Results

### Patient Characteristics

The clinicopathologic characteristics of 105 HGSOC patients are summarized in Table 1. All patients presented with advanced-stage disease consistent with FIGO stage III (71%) or IV (29%) at the time of diagnosis. Forty-two patients (40%) had no platinum-free interval after first line chemotherapy (PFI = 0), while 45 patients (43%) showed complete response and developed relapse at different time points during the follow-up period (PFI > 0). The 45 relapsing patients were included in subsequent analysis by evaluating, for each one, the site of recurrence and the pattern of spread. Most recurrences were abdominal (*n* = 32, 71%) and followed a transcoelomic (*n* = 21, 47%) or mixed (*n* = 12, 27%) route of dissemination. Distant metastases, defined by the presence of lesions in parenchymal organs excluding peritoneal dissemination in the abdominal cavity, occurred in 13 out of 45 relapsing patients (29%) and were mainly hematogenous (*n* = 7, 15%) or lymphatic (*n* = 5, 11%). Metastatic sites of distant recurrences included the liver parenchyma (*n* = 6), brain (*n* = 4), bone (*n* = 1), lung (*n* = 1), and inguinal lymph nodes (*n* = 1).

**Table 1:** Clinicopathological characteristics of 105 HGSOC and 40 normal control patients.

**Characteristics**	**HGSC**	**Normal control**	
		**Hose**	**Tube**
*n*	105	14	26
Age at diagnosis (mean years, range)	63 (23–85)	53 (49–63)	49 (40–63)
**FIGO stage (%)**			
III	75 (71)		
IIIA	9 (12)		
IIIB	3 (4)		
IIIC	63 (84)		
IV	30 (29)		
IVA	10 (33)		
IVB	20 (67)		
**RT (%)**			
0	24 (23)		
>0	77 (73)		
Missing	4 (4)		
**Recurrence**			
Yes	45 (43)		
No	15 (14)		
Never NED^*^	42 (40)		
Missing	3 (3)		
**Site of Recurrence**			
Abdominal	32 (71)		
Distant	13 (29)		
**Spread pattern (%)**			
Transcoelomic	21 (47)		
Lymphatic	5 (11)		
Hematogenous	7 (15)		
Mixed	12 (27)		
**Status**			
Death in relation to malignancy	76 (72)		
Death unrelated to malignancy	6 (6)		
Alive with disease	7 (7)		
Alive without disease	16 (15)		

* *Patients with PFI = 0*.

### Claudin-3, -4, and -7 Expression Is Decreased in HGSOC Compared to Normal Fallopian Tube Epithelia

**Figure 1:**
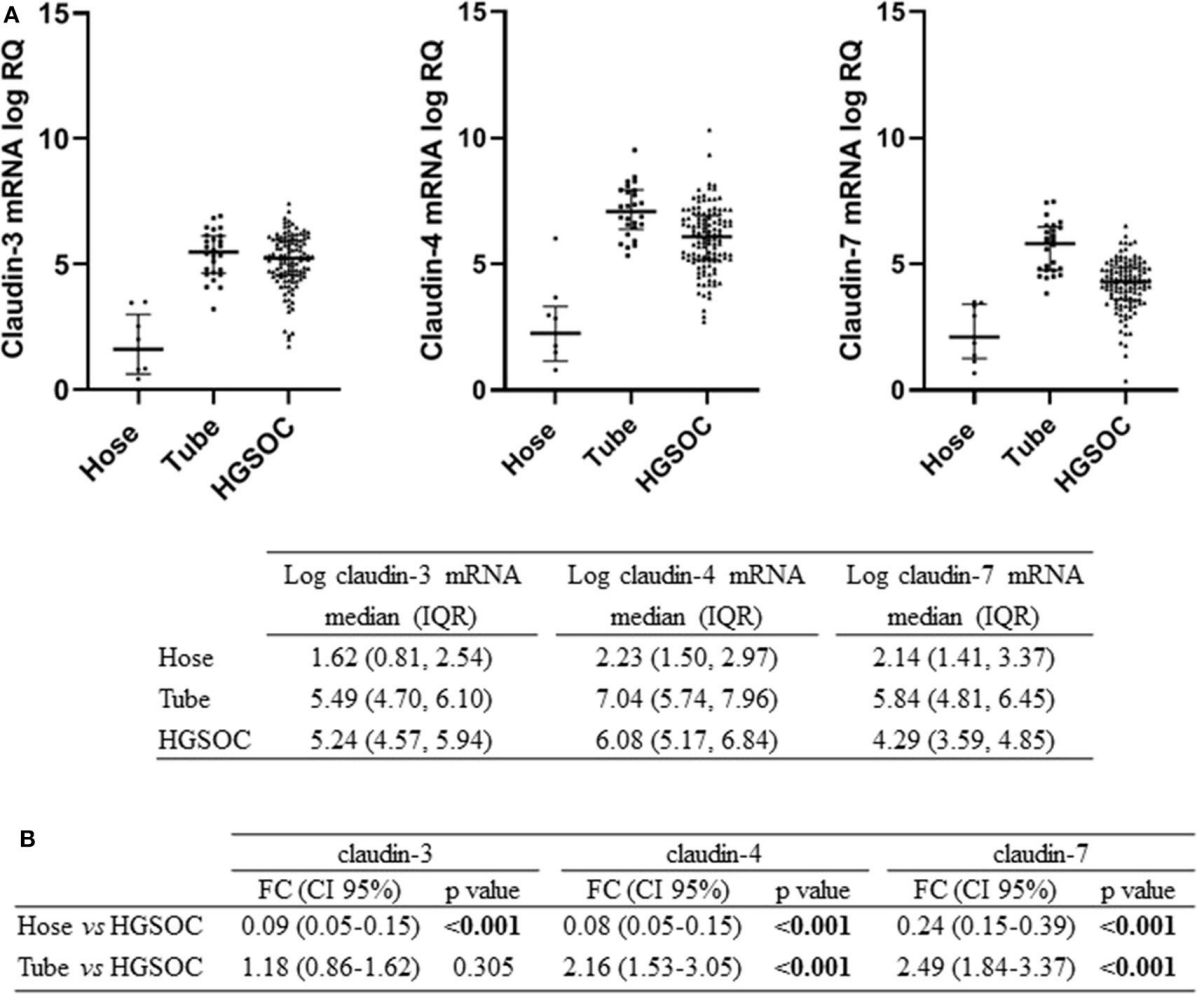
Expression of claudin-3,-4, -7 in normal ovarian surface epithelium (Hose, *n* = 9), normal tube epithelium (Tube, *n* = 26) and ovarian tumors (HGSOC, *n* = 105). **(A)** mRNA expression data are shown as scatter dot plot where the solid lines represent the median with interquartile range (IQR). Value are given as normalized expression relative quantification (log RQ). **(B)** Fold change (FC) is calculated by dividing mean expression in either Hose or tube by the one in HGSOC tissues. Significant comparisons are indicated by bold font.

We tested claudin-3, -4, and -7 mRNA expression in 105 fresh-frozen specimens of HGSOC, 9 Hose, and 26 fallopian tube brushing. All claudins were expressed in tumors and in normal tubal epithelium at variable levels, whereas low to undetectable levels were detected in Hose (Figure 1A). We found gene expression levels of the three claudins highly correlated, with Pearson's r ranging between 0.65 and 0.77 (all *p* < 0.001).

We compared claudin gene expression in HGSOC samples with both Hose and fallopian tubes, the two putative sites of HGSOC origin, obtaining opposite results. When we used Hose as reference tissue, claudins significantly turned out to be upregulated (*p* < 0.001), but markedly downregulated in HGSOC samples if we compared it to normal fallopian tubes (Figure 1B). In particular, claudin-4 and claudin-7 transcript levels were increased by over 2-fold in normal fallopian tubes compared to tumor samples (both *p* < 0.001), whereas claudin-3 showed a 1.18-fold increase in fallopian tubes compared to HGSOCs (*p* = 0.305).

### Low Expression of Claudin-7 in HGSOC Is Associated With Distant Metastases

These results prompted us to investigate the relationship between claudin expression and clinicopathological parameters of HGSOC patients. To this aim, we combined the mRNA expression value of claudin-3, -4, and -7 with weights derived from the first principal component loadings, to generate a new variable called “meta-claudin.” The assigned weights were 0.55, 0.67, and 0.49, respectively for claudin-3, -4, and -7; therefore, meta-claudin can be interpreted as the overall burden of claudin expression.

As shown in Table 2, analysis of meta-claudin suggests a tendency toward downregulation of claudins in tumors stage IV compared to stage III (meta-claudin FC = 0.65, *p* = 0.057). Considering each claudin separately, we found claudin-7 significantly decrease in patients that relapse to distant organs compared to the abdominal recurrence (claudin-7 FC = 0.61, *p* = 0.016), regardless of the initial tumor stage. Moreover, considering the pattern of spread, the diminished expression of claudin-7 remained associated at significant level with the hematogenous diffusion modality (claudin-7 FC = 0.53, *p* = 0.025). No significant association was observed between claudin-3 and -4 expression and site/pattern of metastasis, nor between any claudins and age or residual tumor.

**Table 2:** Correlation between claudin-3, -4, -7 expression and clinicopathological parameters.

**Characteristics**	**Claudin-3**		**Claudin-4**		**Claudin-7**		**Meta-claudin**	
	**FC (CI 95%)**	***p*-value**	**FC (CI 95%)**	***p*-value**	**FC (CI 95%)**	***p*-value**	**FC (CI 95%)**	***p*-value**
**Age (years)**								
> 63 vs. ≤ 63	1.23 (0.92–1.64)	0.154	1.27 (0.94–1.71)	0.119	0.94 (0.72–1.23)	0.648	1.27 (0.84–1.94)	0.257
**RT**								
> 0 vs. 0	1.00 (0.7–1.41)	0.977	1.13 (0.79–1.62)	0.502	1.11 (0.81–1.54)	0.507	1.14 (0.69–1.9)	0.605
**FIGO Stage**								
IV vs. III	0.84 (0.61–1.15)	0.264	0.74 (0.53–1.03)	0.07	0.78 (0.58–1.04)	0.094	0.65 (0.41–1.03)	0.057
**Site of Recurrence**								
distant vs. abdominal	0.99 (0.64–1.53)	0.954	1.05 (0.64–1.71)	0.849	0.61 (0.41–0.91)	0.016^*^	0.8 (0.42–1.55)	0.501
**Spread Pattern**								
Hematogenous vs. Lymphatic	0.65 (0.29–1.43)	0.270	0.88 (0.36–2.1)	0.783	0.58 (0.28–1.19)	0.135	0.55 (0.17–1.78)	0.315
Hematogenous vs. Mixed	0.84 (0.44–1.58)	0.591	0.77 (0.37–1.61)	0.486	0.64 (0.35–1.15)	0.135	0.61 (0.23–1.61)	0.314
Hematogenous vs. Transcelomic	0.78 (0.44–1.40)	0.413	0.88 (0.45–1.72)	0.706	0.53 (0.31–0.91)	**0.025**	0.59 (0.25–1.43)	0.234

* *Corrected for FIGO stage*.

*The association of claudins expression with clinicopathological parameters was tested using linear models. P < 0.05 were considered to be significant and are indicated by bold font*.

**Figure 2:**
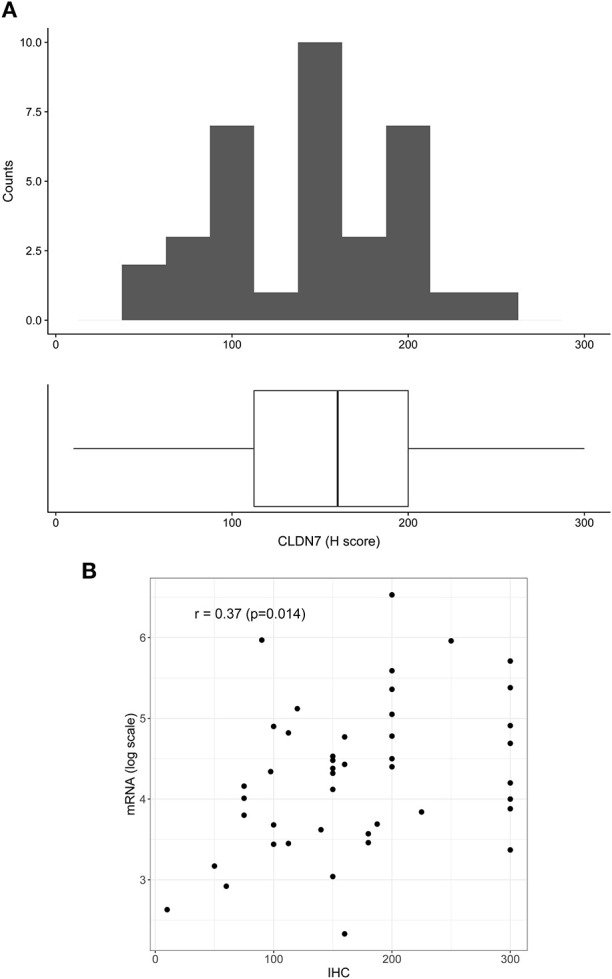
Distribution of H score in the patient positive for claudin-7 immunostaining (*n* = 45). **(A)** The distribution is fairly symmetric with a range going from 10 to 300. Figure shows histogram (upper side) and boxplot (lower side) of claudin-7 H score. **(B)** Scatter plot of claudin-7 mRNA expression values (log scale) and H-score, reflecting increase in H-score associated to a higher mRNA expression (*r* = 0.37, *p* = 0.014).

**Figure 3:**
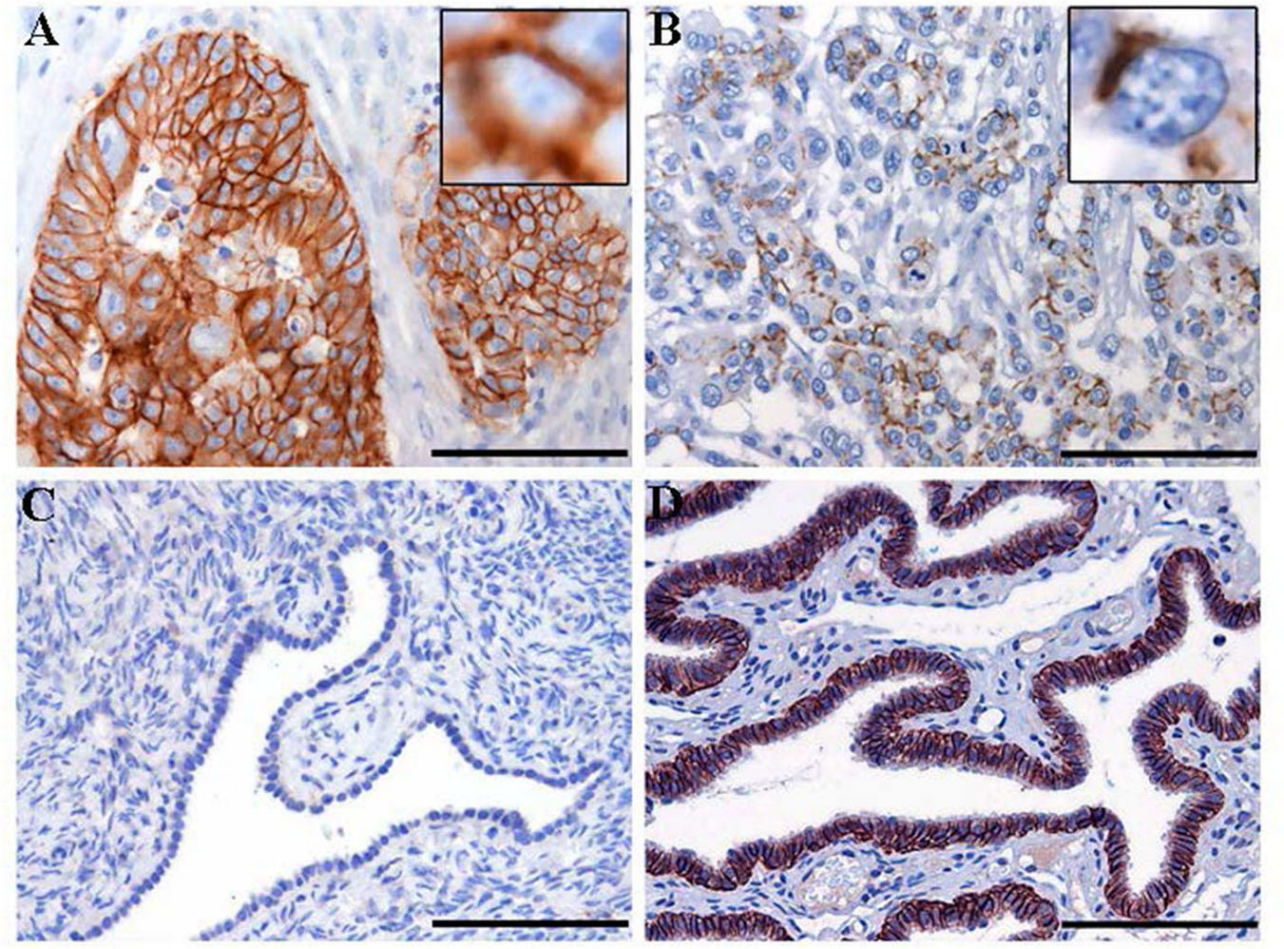
Immunohistochemistry staining for claudin-7 expression in HGSOC tissue samples. Representative cases of primary HGSOC showing heterogeneous spectrum of strong and diffuse **(A)** rather than discontinuous **(B)** membranous staining. Ovarian mesothelium **(C)** and normal fallopian tube epithelium **(D)**, representing respectively the negative and postive control tissue for claudin expression, show absent rather than strong and diffuse membrane staining. Original magnification: 200x (scale bar 100 um).

To confirm mRNA findings at the protein level, we performed claudin-7 IHC staining in the subset of HGSOC patients who developed recurrence. All 45 cases were positive for claudin-7 with a single H score ranging from 10 to 300 (Figure 2A). There was a significant association between H score and mRNA expression, with Spearman's *r* = 0.36 (*p* = 0.014) (Figure 2B), confirming variable expression of claudin-7 protein in cancer samples. We observed two main patterns of membranous staining, the first “homogeneous” with claudin-7 expressed at cell-cell contact as a strong continuous linear pattern and the second “heterogeneous” with discontinuous claudin-7 expression along the cell border (Figure 3). In patients with discontinuous staining, the level of claudin-7 is on average half that of patients with homogeneous pattern (FC = 0.51, *p* = 0.0226).

**Figure 4:**
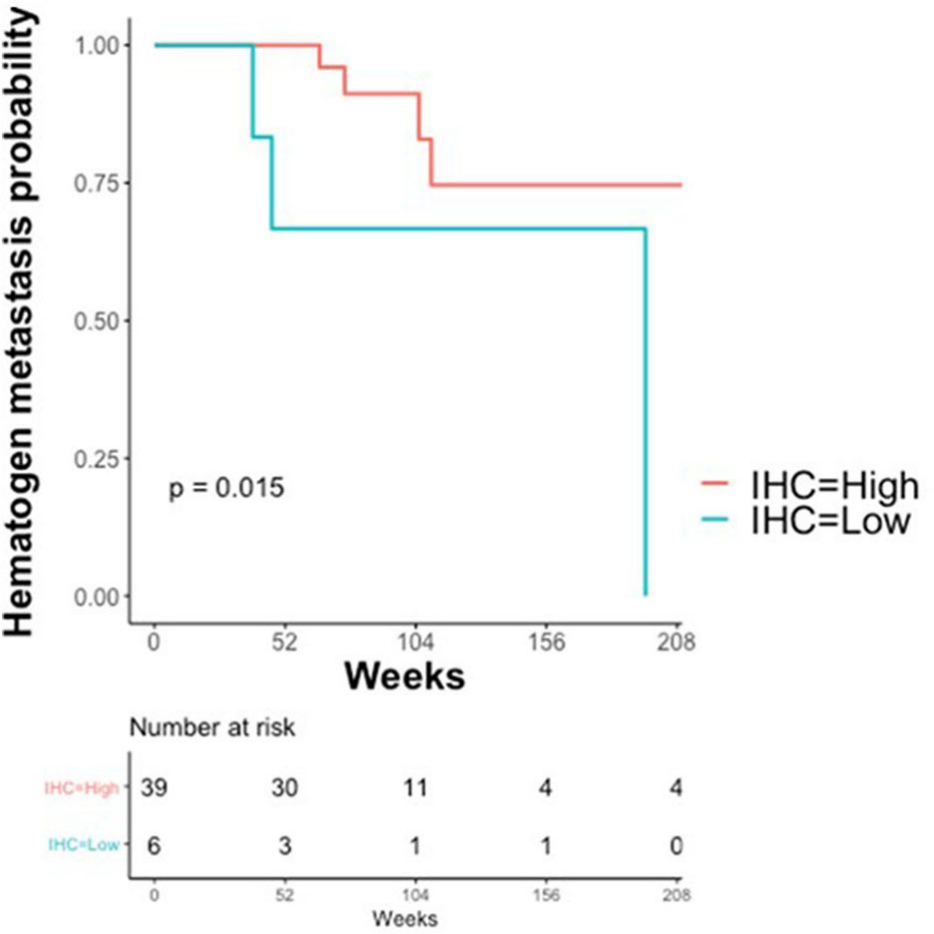
Kaplan–Meier Curves. Kaplan-Meier curves are used to display the relationship between claudin-7 immunohistochemistry score (IHC) and probability of hematogenous metastasis in relapsing patients (*N* = 45). IHC was dichotomized (High vs. Low) using Maximally Selected log-rank statistics.

The association between claudin-7 expression and the risk of hematogenous recurrence was evaluated using a Kaplan-Meier estimator and a log-rank test. An optimal threshold to discriminate high vs. low claudin expression was determined using Maximally Selected log-rank statistics (22). The selected threshold (H-score ≤ 75, unadjusted Log-Rank *p* = 0.015, adjusted *p* = 0.09) suggests that a high risk of developing distant metastases via hematogenous route was associated with very low levels of claudin-7, corresponding to the 10th percentile of the H-score (Figure 4). Notably, 6 out of the 7 cases with hematogenous recurrence and low claudin-7 expression were diagnosed as FIGO stage III, without any clinical or instrumental evidence of distant metastases.

### Downregulation of Claudin-7 Expression Is Indicative of Distant Disease

To evaluate the predictive performance of claudin-7, we fitted a logistic model considering patients with distant metastases, either at presentation or at recurrence, restricting our cohort to patients that metastasize exclusively through hematogenous route. Accordingly, we included 7 recurrent and 20 stage IVB patients. Stage IVA patients were not taken into account, as malignant pleural effusion is more likely to result from the pleural invasion from contiguous structures, such as the diaphragm, or from the transdiaphragmatic migration of malignant cells thorough pleuro-peritoneal communications, rather than from hematogenous dissemination (23).

**Figure 5:**
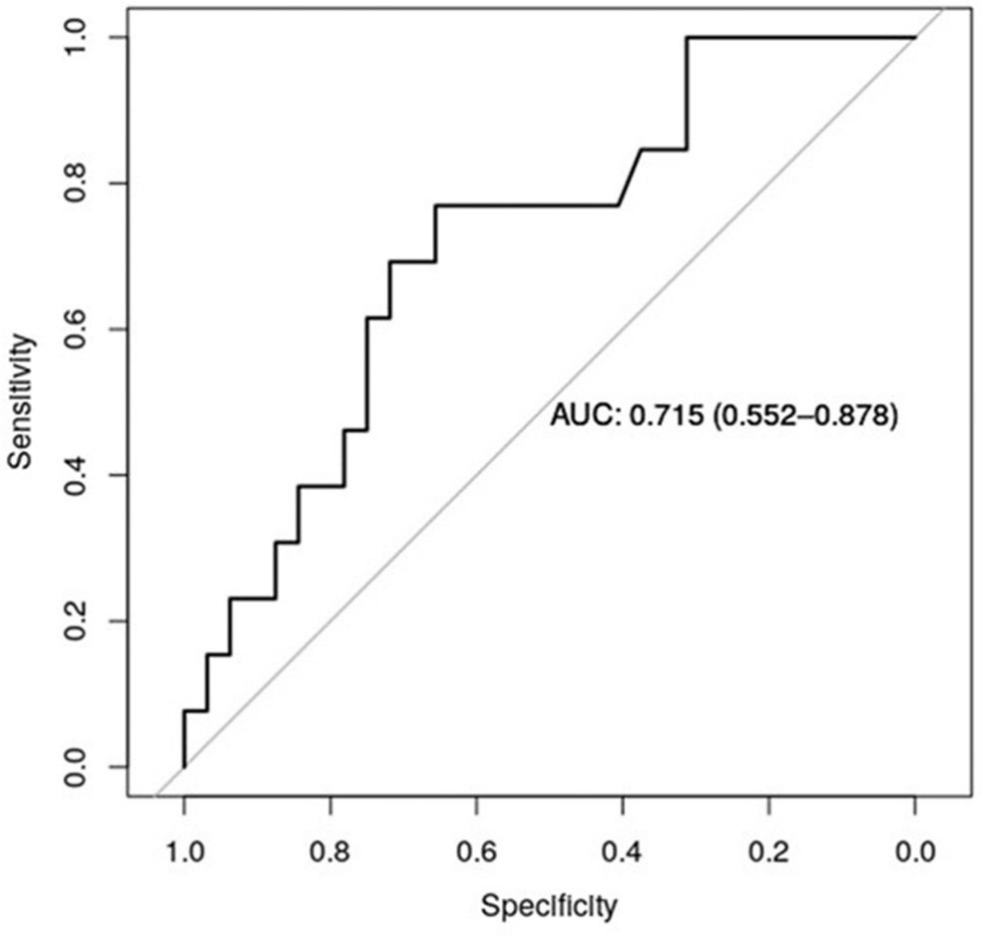
ROC curve for claudin-7 and site of metastases. The figure show the ability of claudin-7 mRNA to distinguish patients with distant hematogenous metastases from transcoelomic/lymphatic metastases. The calculated AUC is 0.715 (95% CI, 0.55–0.88).

Figure 5 shows the receiver operator curve (ROC) for claudin-7 and site of metastases, with the corresponding AUC being 71.5% [CI_95%_ 55.2–88.7%]. Furthermore, using a logistic regression model, we estimated the reduction in the probability of distant disease of 39% per unit increase in the level of claudin-7 (*p* = 0.03). The AUC computed from the ROC curve is inherently optimistic, therefore we computed the optimism-adjusted C-index (concordance index, numerically equivalent to the AUC) from the logistic model to be 65.9% [CI_95%_ 53.2–76.4%] (24). These data suggest a potential role of claudin-7 in discriminating distant metastatic events.

### Gene Sets Enriched in Distant Metastatic HGSOC Patients

**Figure 6:**
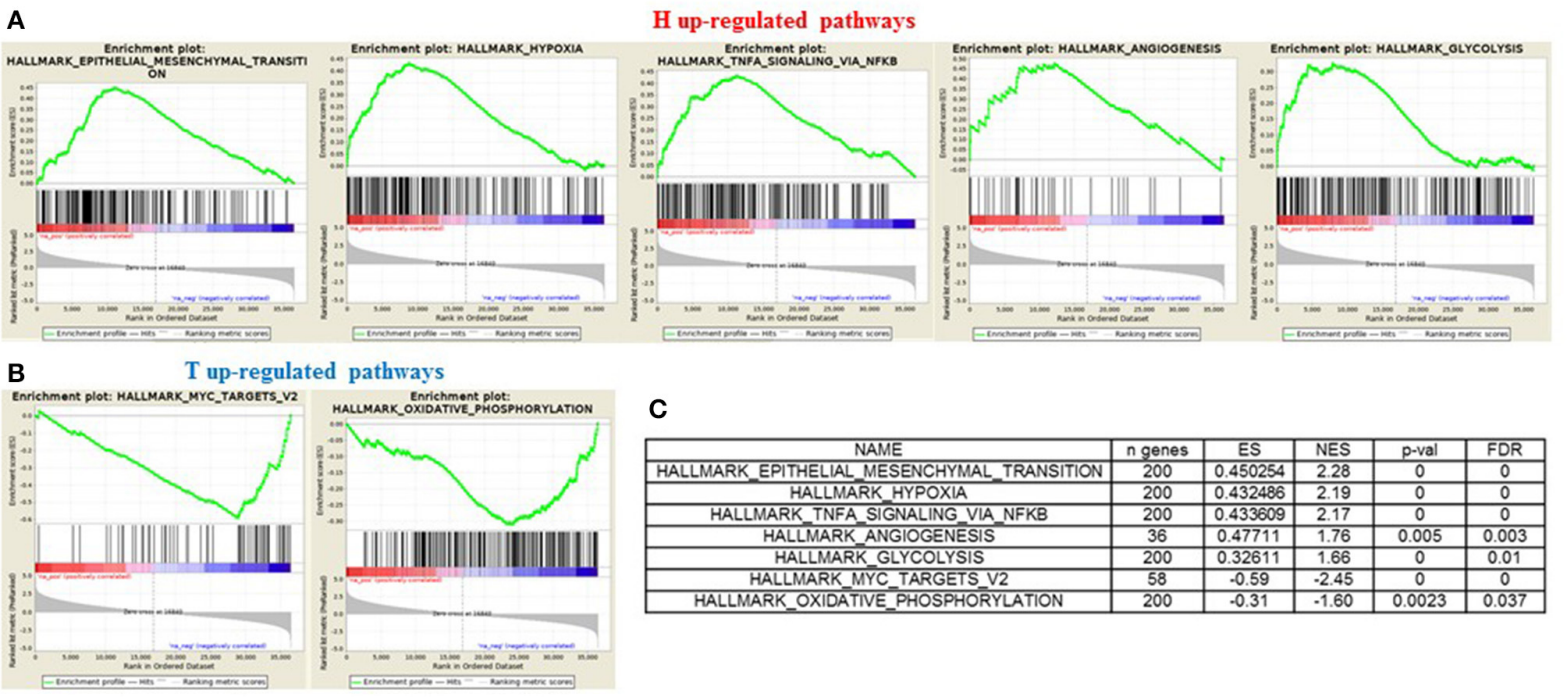
GSEA “H phenotype” vs. “T phenotype.” Enrichment score plot of EMT, Hypoxia, Angiogenesis, NFKB, Glycolysis terms **(A)** and myc_targets_v2, oxidative_phosphorylation terms **(B)** positive enriched in hematogenous or transcelomic patients group. The plots contain the profile of the running enrichment score (ES-green line) and positions of gene set members on the rank ordered list in GSEA (black strokes on the bottom). **(C)** GSEA result summary.

In order to gain insights into the underlying biological mechanism of HGSOC hematogenous dissemination, we applied GSEA to the HGSOC gene expression profiles, focusing on two groups of recurrent patients presenting distinct pattern of spread. A total of six patients with hematogenous recurrence (referred to as “H phenotype”) and 20 patients with transcoelomic recurrence (referred to as “T phenotype”) were profiled for gene expression using Agilent G4851B microarray, and further included in the enrichment analysis. Nine HALLMARK gene sets result significantly enriched (FDR < 0.1) in the H phenotype compared to T phenotype (Table S1), where the top 3 are related to *EMT, Hypoxia*, and *NFKb* signaling pathways **(**NES 2.28, 2.19, 2.17, respectively). Furthermore, *glycolisis* and *angiogenesis* terms result strongly associated to hematogeneous phenotype (Figures 6A–C), emphasizing the nature of this class compared to transcoelomic patients characterized by an enrichment of *myc_targets_v2* and *oxidative phosphorylation* gene sets (Figures 6B,C and Table S1). Interestingly, angiogenesis results physically connected by APP and COL3A1 genes with glycolysis pathway, defined in turn by the presence of CLDN3 and CLDN9 in its signaling, suggesting a possible role of claudins in the vascularity process of hematogeneous recurrent patients (Table S2).

## Discussion

In the context of epithelial cancers, the loss of cell adhesion is regarded as a prerequisite for cancer progression to a metastatic phenotype. The extent of tight junction (TJ) formation is a key factor that regulate motility and invasion, and maintenance of TJ requires claudin expression and integrity. Accordingly, a decrease or loss of claudin expression has been documented in various human malignancies (25). The reported overexpression of claudin-3, -4, and -7 in ovarian tumors suggests, on the contrary, a cancer-promoting role that is largely unexplained. None of these studies showed a clear association between the clinicopathologic characteristics of the tumor or survival and overexpression of claudins, whose prognostic significance remains controversial (26, 27).

This can be partly be explained by the fact that these studies consider mixed cohorts of ovarian cancers irrespective of histology and of tissue of origin, while it is now established that the histological types of ovarian carcinomas are different entities with divergent molecular profiles and clinical outcomes (28). This finding has a strong impact on comparative gene expression profiling studies, where the genes identified as differentially expressed in tumor samples depends on the choice of the control tissues used as a source of normal epithelium.

In the present study we evaluated claudin-3, -4, and -7 expression in our large and homogeneous cohort of 105 HGSOC fresh-frozen tissues and compared it with either the expression in normal ovarian or in fallopian tubal epithelium, both currently considered potential source of such tumors. To our knowledge, this is the first analysis of claudin mRNA expression in tubal epithelium from healthy individuals. Unlike the ovarian surface epithelium, which consistently show no claudin staining (13, 29), tubal epithelium expresses high levels of either claudin proteins (30). Here we demonstrated that: (i) the overall expression level of claudin-3, -4, and-7 mRNA is lower in HGSOC samples than in tubal epithelium; (ii) HGSOC display a wide range of claudin expression, with a small fraction having a particularly low-level of expression. A claudin-low molecular subtype has been shown to occur in other epithelial tumors, such as breast (31), bladder (32), and gastric cancers (33). In addition to low expression of claudins they show enrichment of EMT transcription factors. A unique feature of this tumor subtype is the lack of cell-cell junctions, characterizing the EMT process that governs metastatic dissemination.

In the present study claudins expression tended to be reduced in HGSOC samples from patients with distant metastatic disease at diagnosis (FIGO stage IV) and in patients with extra-abdominal recurrence. This relationship was particularly evident for claudin-7, which showed the strongest downregulation in patients who relapsed to distant organs and was significantly associated with a hematogenous pattern of spread. In addition, in patients with reduced expression of claudin-7 assessed by IHC, we observed a pattern of discontinuous membranous staining indicative of a discohesive architecture that can trigger the release of tumor cells from the primary site. These tumors may therefore already have the intrinsic capacity to invade the bloodstream and metastasize at distance. Indeed, according to functional enrichment analysis, the most represented biological pathways in primary HGSOC samples of patients with distant metastases compared to patients with abdominal disease were associated to EMT, angiogenesis, hypoxia, and glycolysis. These are closely interrelated processes that cooperate to promote tumor proliferation and dissemination (34).

Collectively, our data are consistent with a possible involvement of claudin-7 in ovarian cancer progression and, in the literature, the most recent publications support the pro-metastatic role of a diminished expression of claudin-7 in different type of epithelial cancers. For instance in colorectal cancer, where claudin-7 downregulation promotes the invasion and metastasis by regulating the EMT process (35), and in renal cancer, where downregulation of claudin-7 via hypermethylation of its promoter has been demonstrated and associated with metastatic features *in vitro* and *in vivo* (36).

Ovarian cancer metastasize primarily by intraperitoneal dissemination with direct exfoliation of epithelial cells throughout the peritoneal cavity and retroperitoneal lymphatic invasion. Hematogenous metastases are unusual at presentation and considered as a late event in the clinical evolution of the disease (37). However, some authors have reported a slight, but significant, increase in the incidence of brain and hepatic involvement over the last years, likely due to an improvement in the control of intra-abdominal disease: the number and type of previous treatment in fact, demonstrated influence on the relapse pattern in ovarian cancer patients. This observation is supported by Robinson et al., who reported that women treated with intraperitoneal chemotherapy after optimal debulking surgery are at high risk of extraperitoneal metastases, including central nervous system (CNS) and skin (38). Moreover, Rauh-Hain et al. demonstrated a higher rate of extra-abdominal disease in patients treated with antiangiogenic agents, specially pleura and lung (39). An improvement in intra-abdominal control of the disease may lead to an increase of distant metastases incidence thanks to prolongation in survival and modification of pattern of spread.

While the translational impact of this finding is currently limited by the lack of treatments that prevent distant metastasis, the identification of molecular biomarkers that can discriminate at diagnosis tumors that can potentially spread at distance is of the utmost importance, for a better prognostic classification of the disease and to further tailor long-term surveillance, particularly with regard to the risk of CNS metastasis.

A limitation of this study is given by the small number of patients with hematogenous metastasis, which, however, reflects the low incidence of this clinical presentation in HGSOC. Moreover, the validation of biomarkers associated with distant/hematogenous metastasis is a critical issue, since the information regarding site and pattern of metastatization is lacking in the literature, even in the largest public databases such as TCGA o Curated Ovarian Data. This study is the first step toward unraveling a potential mechanism underlying distant (hematogenous) metastasis in HGSOC. Claudin-7 expression is worthy to be further investigated in prospective studies on larger cohort of tissue samples either to test its potential clinical utility in late-stage HGSOC or to assess its value for metastasis prediction in early-stage disease.

## Data Availability Statement

The datasets generated for this study can be found in the EMBL-EBI Arrayexpress repository: https://www.ebi.ac.uk/arrayexpress/experiments/E-MTAB-7083/ and https://www.ebi.ac.uk/arrayexpress/experiments/E-MTAB-7084/.

## Ethics Statement

The studies involving human participants were reviewed and approved by Research Review Board-the Ethic Committee- of the ASST Spedali Civili, Brescia, Italy. Study reference number: NP1676. The patients/participants provided their written informed consent to participate in this study.

## Author Contributions

CR, VZ, EB, and SC conceived and performed all of the experiments, analyzed the data, and wrote the paper. MS, MC, LA, MB, PT, LZ, and AR contributed to the analyses and interpretation of data. SMa, MD'I, FF, AG, FO, ES, AS, and SMi contributed to paper discussion and review of the manuscript. All authors contributed to the article and approved the submitted version.

## Conflict of Interest

The authors declare that the research was conducted in the absence of any commercial or financial relationships that could be construed as a potential conflict of interest.
