# Supplementary figures and images for: Low Expression of Claudin-7 as Potential Predictor of Distant Metastases in High-Grade Serous Ovarian Carcinoma Patients
Source: Front Oncol. 2020 Aug 4;10:1287. doi: 10.3389/fonc.2020.01287 (PMC7417514; doi:10.3389/fonc.2020.01287)

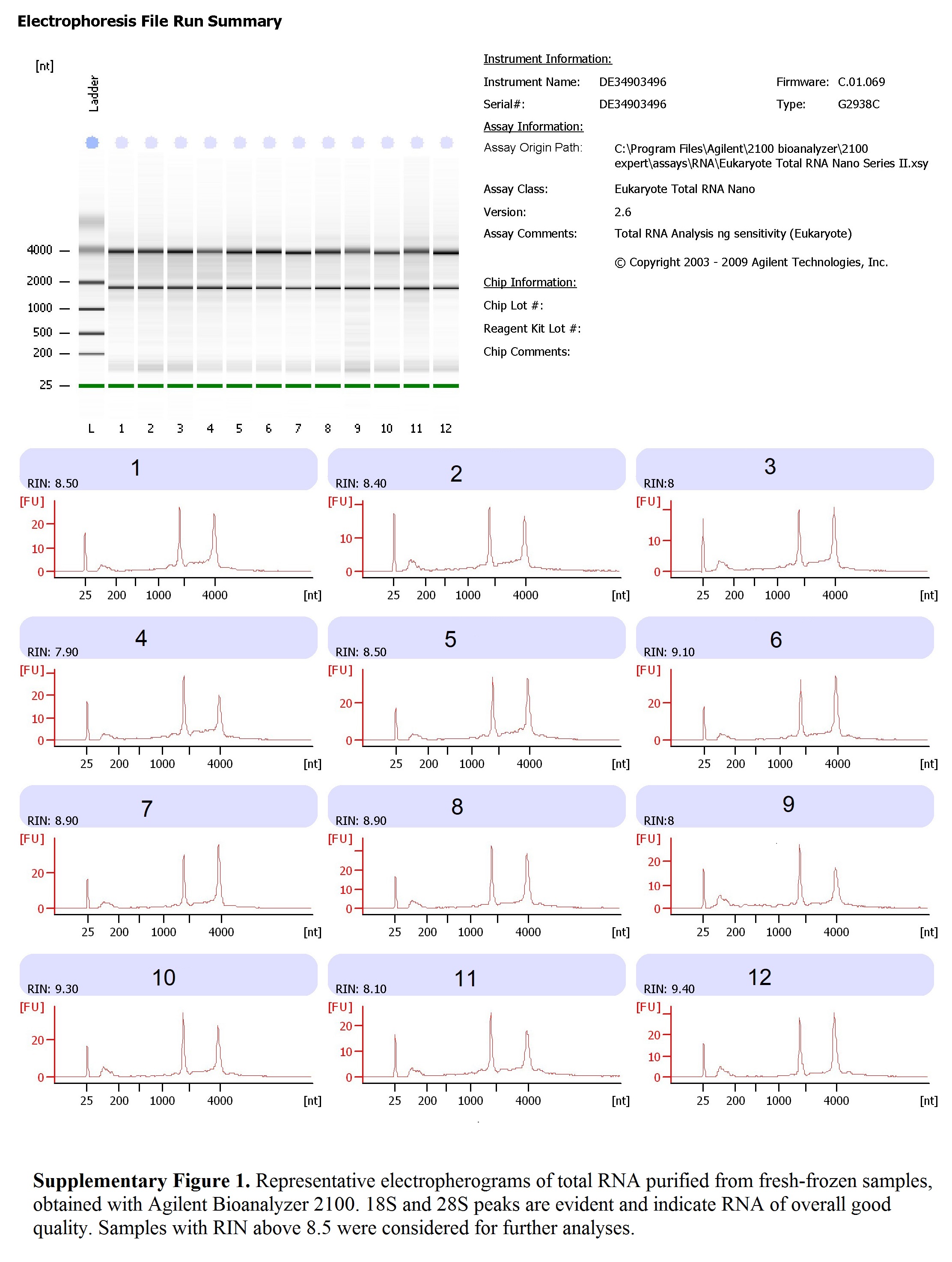

Supplement: Supplementary file 1 [file Image_1.JPEG]
